# Evaluating the Effectiveness of a School-Based Mental Health Training Programme: The Transformative, Resilient, Youth-Led (TRY) Gym

**DOI:** 10.3390/healthcare14010009

**Published:** 2025-12-19

**Authors:** Wai-Chung Chung, Fan Jiang, Yin Ling Beryl Fok, Cheung Ying Chiu, Winnie Wing Yan Yuen, Josephine Wing-Fun Fung, Anson Chui Yan Tang, Po Fai Jonah Li, Raymond Chi-Fai Chui, Chi-Keung Chan

**Affiliations:** 1 School of Arts and Humanities, Tung Wah College, Hong Kong SAR, China; alvinchung.ca.ca@gmail.com (W.-C.C.); fjiang@twc.edu.hk (F.J.); berylfok@twc.edu.hk (Y.L.B.F.); 2 Department of Social Work, Hong Kong Shue Yan University, Hong Kong SAR, China; cychiu@hksyu.edu (C.Y.C.); wffung@hksyu.edu (J.W.-F.F.); cfchui@hksyu.edu (R.C.-F.C.); 3 Department of Counselling and Psychology, Hong Kong Shue Yan University, Hong Kong SAR, China; wyyuen@hksyu.edu; 4 School of Nursing, Tung Wah College, Hong Kong SAR, China; ansontang@twc.edu.hk; 5 Department of Social Sciences, School of Interdisciplinary Arts and Sciences, University of Washington Tacoma, Tacoma, WA 98402-3100, USA; jonahli@uw.edu; 6 Translational Research Centre for Digital Mental Health, Tung Wah College, Hong Kong SAR, China

**Keywords:** mental health, youth-led, school-based, co-creative, transformative, resilient and stigma-free

## Abstract

**Background**: The Transformative, Resilient, and Youth-Led/Driven (TRY) Gym, a school-based co-creative mental health training programme, is grounded in the Positive Youth Development (PYD) approach. It seeks to improve adolescents’ mental health and well-being by strengthening their resilience and competence. Additionally, it prepares them to deliver peer-led mental health activities, promoting mental wellness and fostering a stigma-free, supportive environment. **Methods**: This study evaluated the programme using a mixed-method design. In total, 94 students from eight secondary schools in Hong Kong were recruited, with 80 participating in the evaluation. **Results**: Five outcomes showed significant improvements from baseline to the post-implementation phase, including social competence, cognitive competence, emotional competence, resilience, and mental well-being. Common features were identified across interviews, which may possibly account for the significant results and participants’ improved mental health. **Conclusions**: The TRY Gym programme’s fidelity was demonstrated by its completion, which included high participant responsiveness, and a co-creative and youth-driven approach in the project. In addition, the positive outcomes of the programme underscore its effectiveness in improving mental health among adolescents by imparting mental health knowledge and providing opportunities for participants to apply learnt techniques in everyday life situations.

## 1. Introduction

The mental health of adolescents is a growing concern both globally and locally in Hong Kong, demanding urgent attention and action. According to the World Health Organization [1], one in seven (14%) 10–19-year-olds worldwide experience mental health conditions, contributing to 15% of the global disease burden for this age group, with suicide being the third leading cause of death among those aged 15–29. In Hong Kong, the situation is equally alarming, as outlined in the Youth Development Blueprint [2], which highlights that the fast-paced, high-pressure, and crowded environment fosters significant mental health challenges for young people. The deterioration of teenagers’ mental well-being is closely linked to an increase in suicidal behaviours, with 228 student suicides reported from 2014 to 2024, peaking at 32 cases in 2023 and slightly declining to 28 in 2024, still the second-highest on record [3,4]. A Department of Health study [5] found that 1.7% of secondary students and 2.8% of primary students planned suicide, while 0.8% of secondary and 1.3% of primary students attempted it in the 2023/24 school year, based on a sample of approximately 257,000 primary and 173,000 secondary students. Furthermore, the Hong Kong Suicide Press Database (HKSPD) reported 11 press-covered suicide cases among 11–16-year-olds from January to mid-April 2025 [6], and 24.4% of 6082 children aged 6–17 surveyed suffered from at least one mental disorder, with half experiencing multiple disorders [3,7]. These alarming trends underscore the critical need to review and enhance existing mental health support systems and policies while adopting innovative strategies to address the mental health crisis among adolescents globally and in Hong Kong.

### 1.1. How Schools, Family, and Characteristics of Adolescent Developmental Stage Influence Academic Stress in Secondary School Students

Academic stress is a significant concern for secondary school students, particularly in high-pressure educational environments like Hong Kong, where academic achievement is highly valued. This section explores how familial and school-related factors contribute to academic stress, drawing on relevant theories and empirical evidence to inform the development of a mental health training programme for secondary school students. Additionally, it examines how these factors interact to amplify academic stress, providing a comprehensive understanding of their combined effects.

#### 1.1.1. Familial Factors and Academic Stress

Familial factors play a crucial role in shaping students’ academic stress levels, as they influence emotional, behavioural, and academic outcomes through daily interactions and expectations [8,9,10,11]. The family process model suggests that economic stress and the healthiness of couple relationships would affect the well-being of parents and parent–child relationships. Poor parent–child relationships might trigger intergenerational conflict that affects children’s behavioural, emotional, and academic adjustment adversely [11,12]. Similarly, attachment theory highlights that the quality of the parent–child relationship affects students’ emotional and cognitive outcomes, which can influence their academic stress levels [13,14,15].

The emotional bond between parents and children significantly impacts academic stress. In East Asian cultures, such as Hong Kong, a close parent–child relationship is often associated with higher academic stress [9,16]. This occurs because strong familial bonds may increase students’ sense of obligation to meet their parents’ high academic expectations, leading to greater pressure to perform [9,16]. For example, when students feel closely connected to their parents, they may internalize parental academic goals, which can amplify stress [9,17], particularly in the context of public examinations. This contrasts with findings from Western societies, where a supportive parent–child relationship is typically linked to reduced academic stress due to open communication and emotional support [18,19].

Parental expectations for academic success, or parental achievement aspiration, are prevalent in East Asian societies since academic success is perceived as a familial obligation [17,20,21,22,23]. Research suggests that parents’ attitudes toward academic attainment contribute to academic stress [24,25,26]. In cultures influenced by Confucian values, academic achievement is seen as a means to bring honour to the family, placing additional pressure on students [21]. Empirical studies have shown that when adolescents perceive strong parental emphasis on academic performance, they experience increased stress, particularly when they feel their achievements are tied to pleasing their parents [10,24,27]. This pressure is especially pronounced in Hong Kong, where parental expectations can impose a substantial burden on secondary school students preparing for public examination [27].

#### 1.1.2. School Factors and Academic Stress

The school environment is another critical determinant of academic stress, as it shapes students’ well-being, academic stress, and adjustment [28,29,30]. Theories such as the social setting theory and stage-environment fit theory emphasize that the school context, including the learning atmosphere and interpersonal interactions, significantly affects students’ mental well-being and academic stress levels [31,32,33,34].

School climate, which encompasses norms, interpersonal relationships, and teaching practices, plays a pivotal role in students’ academic experiences [35]. A positive school climate fosters psychosocial well-being, behavioural appearance, and academic achievement and stress [33,36]. Conversely, a negative school climate, characterized by a mismatch between students’ needs and the opportunities provided by the school, can lead to maladjustment and increased academic stress [32,33]. In East Asian contexts, where curriculum demands and class sizes differ from Western systems, the school climate may uniquely contribute to academic stress due to discrepancy between educational design and setting [37]. The school climate was an insignificant predictor of academic stress in an empirical study from Hong Kong [38].

The emphasis on academics in schools, defined as the pressure exerted by schools to meet high academic standards, is a significant contributor to academic stress [39]. In East Asian schools, where academic performance is often prioritized, students face expectations to work diligently and achieve excellence [39,40]. These expectations can lead to increased effort but also result in negative outcomes, such as feelings of inadequacy and academic stress [41,42]. Empirical studies suggest that a highly demanding academic environment, characterized by comparisons and high standards, can exacerbate stress among secondary school students, or result in mental problems like depression and a sense of inadequacy [41,42].

#### 1.1.3. Characteristics of Adolescent Developmental Stage

Adolescence is a developmental stage that begins with puberty and ends at the onset of adulthood [43]. During this period, individuals undergo significant neural, hormonal, and interpersonal changes [44]. One key development is the enhanced plasticity of the hypothalamic-pituitary-adrenal (HPA) axis, which regulates stress responses [44]. This increased plasticity makes adolescents more susceptible to stress as well as both positive and negative environmental influences. Research suggests that the recalibration of stress reactivity may be facilitated by this heightened plasticity, making adolescence a second critical period—after early childhood—during which stress responses can be reshaped [45,46,47,48,49]. Additionally, adolescence is a crucial time for brain development. Neural plasticity is heightened, leading to greater sensitivity to environmental exposures [50]. This period is particularly important for the association cortex, and higher-order cognitive and emotional processes [51]. Beyond physiological changes, adolescence also marks a shift in well-being priorities—from family-oriented connections to peer relationships [52,53,54].

The social ecology model highlights the interconnectedness of individuals and their surrounding environment [55]. Research indicates that perfectionism, social-oriented achievement motivation, parental achievement aspiration, parent–child relationships, and emphasis on academics in school are significant predictors of academic stress [38]. For secondary school students, their academic stress and psychological traits are results of interaction among characteristics of individual, family, and school. Moreover, regarding the characteristics of the developmental stage of adolescence, the impact from school and family might be amplified for their sensitivity to environmental exposures, especially stress.

### 1.2. Response from Government—Three-Tier School-Based Emergency Mechanism

In 2023, the Hong Kong government developed and implemented the current “Three-Tier School-based Emergency Mechanism” in all the secondary schools of Hong Kong, which provides a structured framework and guidelines for adolescents and youth workers (i.e., teachers, school social workers, and professionals) dealing with students’ mental health issues. The first tier is to assist schools to identify students at an early stage with higher suicide risk or mental health needs. The second tier is to organize an off-campus support network through cross-departmental, cross-professional, and cross-sectoral co-operation to enhance external support for schools in the short term. For the third tier, school principals can refer students with severe mental health needs to the psychiatric specialist services of the Hospital Authority (HA) [56]. However, the mechanism is not without limitations. First, there is a lack of interconnection between tiers, which results in students being assigned to an inappropriate level without adequate support. Professor Paul Yip, the Director of the Hong Kong Jockey Club’s Centre for Suicide Research and Prevention at the University of Hong Kong, discussed the overuse of tier three (transferal of students to mental health services in public hospitals) due to a lack of psychological counselling from experts when at-risk students were discovered in schools by teachers or social workers. Furthermore, the high turnover rates of school teachers and social workers due to migration and seeking better benefits have also intensified the lack of significant others in long-term caring for students. The overzealous help for students who only displayed minimal symptoms or issues has led to a heavy workload on hospital-based mental health services, and also a long wait period for adolescents in need of services [57]. In addition, the current three-tier system fails to identify adolescents who impulsively attempt or commit suicide without seeking help. Research indicates that adolescents often avoid seeking assistance due to self-reliance, stigma, embarrassment, and poor mental health literacy [58,59]. The ineffectiveness of the current programme can be reflected from the sustained, high student suicidal rates recorded in recent years. In 2024, 28 students died from suicide, the second highest among the last 10 years [4,60]. Also, according to Yeong [6] from Hong Kong Suicide Press Database (HKSPD), 11 suicide cases were reported by the press who aged from 11 to 16, within January to mid-April in 2025. This highlights the system’s inability to detect and assist students suffering silently, underscoring the need for new programs to enhance adolescent mental well-being in Hong Kong. However, the evaluation of the Three-Tier School-based Emergency Mechanism is still in progress [61].

### 1.3. Preventive School-Based Mental Health Programmes

To address gaps in current policies, prioritizing a preventive approach could be more effective. While schools and families significantly influence adolescent stress and mental health, changing their dynamics is challenging due to entrenched social norms and the need for governmental coordination. Nevertheless, there is an urgent need for innovative solutions to enhance adolescents’ overall mental health. Primary prevention acts at the stage of pre-pathogenesis, that is, when the disease is yet to occur [62]. Mental health promotion prevents the onset of mental illnesses by reducing risk factors in an individual and strengthening protective elements in them [63]. Representing the second social environment in which adolescents spend considerable time, schools present numerous opportunities and challenges for student development, rendering it an appropriate setting for intervention [64]. This recognition has led to the development of preventative school-based mental health programmes that provide young people with basic knowledge about mental illnesses, promoting resilience and mental well-being among adolescents [65,66].

Nevertheless, most current school-based programmes have similar drawbacks of having a provider-led manner and a top-down approach, which lead to mixed outcomes. A case in point would be the universal school-based programme of “Little Prince is Depressed” (LPD). The follow-up study revealed a nonsignificant result in improving depressive symptoms, with the teacher-led group outperforming the professional-led group in terms of lowering students’ tension and anxiety [67]. Considering that adolescents are in a stage of seeking independence and displaying opposition to authority-driven activities, resistance to authority could be a potential disadvantage of provider-led programmes [68]. Furthermore, the top-down approach has been criticized for a lack of involvement and empowerment among adolescents. When programmes are imposed from above rather than developed with their input, adolescents may feel less engaged and invested. This can reduce the effectiveness of initiatives aimed at empowering adolescents to take ownership of their mental health [69].

To provide broader international context, similar challenges and innovations are evident in school-based mental health interventions in other high-pressure non-Western educational systems. For instance, the HASHTAG (Health Action in ScHools for a Thriving Adolescent Generation) programme, co-designed and implemented in Nepal and South Africa, addresses adolescent mental health in low- and middle-income countries (LMICs) marked by poverty, trauma, and limited resources [70]. Like TRY Gym, HASHTAG adopts a multi-component, whole-school approach, incorporating evidence-based elements such as emotional regulation, stress management, mindfulness, problem-solving, interpersonal skills, assertiveness training, and alcohol/drug education. It targets adolescents aged 12–15, emphasizing resilience and preventive strategies through classroom sessions (Thrive Together) and school-wide activities (Thriving Environment in Schools), including teacher well-being modules to foster supportive environments.

Comparing TRY Gym with HASHTAG highlights cross-cultural insights: both programs prioritize skill-building and resilience in resource-constrained settings, with transferable features like emotional self-efficacy and peer relationships that align with Positive Youth Development (PYD) principles. However, HASHTAG’s focus on co-design with stakeholders (e.g., adolescents, teachers, and parents) in trauma-affected contexts contrasts with TRY Gym’s youth-driven, transformative model in Hong Kong’s academically intense environment, where youth leaders co-create content and engage with recoverees via a “human library” to reduce stigma. This youth empowerment in TRY Gym addresses top-down limitations more directly, potentially offering a transferable innovation for LMICs like Nepal and South Africa, while HASHTAG’s whole-school integration could enhance TRY Gym’s scalability in high-pressure Asian systems. Such comparisons underscore the Hong Kong model’s unique emphasis on self-transcendence and prosocial behaviours, adaptable across cultures to promote sustainable mental well-being.

Furthermore, contemporary school-based programmes advocate for a paradigm shift, wherein educators must adopt an expanded perspective of their duties and responsibilities to address adolescents’ mental health issues at schools. Such an increased workload has resulted in a reluctant attitude from teachers towards leading mental health programmes at schools. The pilot School Mental Health Support Scheme (SMHSS) review found that teachers were hesitant to broaden their roles to include teaching the whole person or managing their students’ mental health issues since they had already been overworked and in a competitive position to get their students good grades [64]. This indicated that school-based programmes with a provider-led, top-down approach had increased teacher burden, creating a significant manpower challenge that further impacted the long-term viability of the programmes. Considering the gaps in current programmes, the TRY Gym programme, an acronym for Transformation-focused, Resilient-promoting, and Youth-driven, was developed by integrating the Positive Youth Development (PYD) framework with contemporary advancements in positive psychology [71].

### 1.4. The Theoretical Framework Underlying the TRY Gym Programme

**Figure 1:**
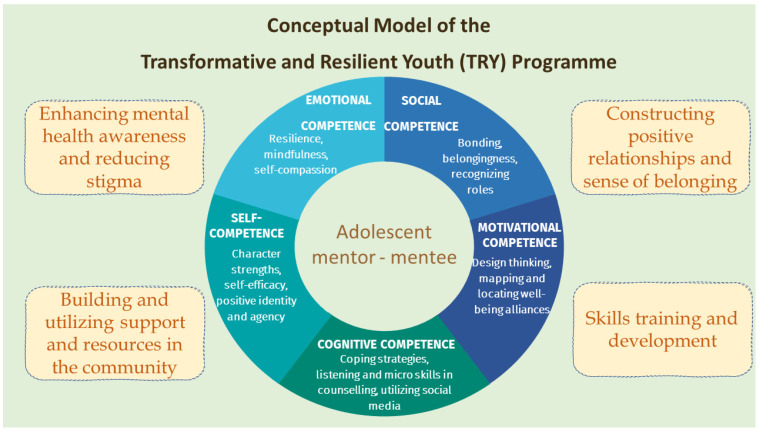
Conceptual model of TRY Gym.

The present programme took the perspective from the Positive Youth Development (PYD) approach, which regards developmental resources and adolescent potential as protective factors for mental well-being. While PYD could be conceptualized as internal (e.g., positive identity), external (e.g., social support), and social assets (e.g., social awareness) [72,73], a review of effective PYD programmes identified 15 important PYD constructs that should be promoted [73]. All young people have strength, as evidenced by their capacity to develop and change in the cognitive, emotional, social, and self aspects during adolescent years [74]. When these strengths are nurtured by supportive measures and environments at home, in schools, and within communities, the positive development of youth, further to their mental health, can be improved [75]. These findings have supported the PYD approach and challenged the past “deficit model” of adolescence, which once framed adolescents’ problems as things to be fixed rather than potentials to be cultivated. Hence, the TRY Gym was based on the conceptual model below (see Figure 1), focusing on five core competencies including emotional, cognitive, social, motivational, and self-competencies, as well as personal resilience, self-efficacy, and bonding to enable young people to identify their strengths and facilitate them to grow, learn, and transform. Recent research underscores the importance of emotional self-efficacy in moderating empathy’s role in educational settings, particularly for fostering inclusive and supportive environments. For instance, Graziano et al. [76] found that higher empathy enhances teachers’ self-efficacy in inclusive education when emotional self-efficacy (the ability to regulate negative emotions like stress or frustration) is high, with stronger effects among females. In TRY Gym, this informs us of our emphasis on building emotional self-efficacy among youth leaders, enabling them to empathically support peers while avoiding emotional overload, thus promoting sustainable resilience and mental well-being in school contexts.

Alongside the PYD perspective, theories and practices of Positive Psychology 2.0 (PP 2.0) were embedded in the TRY Gym programme. Extensive research has identified the updated four pillars of PP 2.0: virtue, meaning, resilience, and well-being, emphasizing that it is difficult for people to survive and flourish without any of these four ingredients [77]. These pillars guided the development of the TRY Gym programme’s content, with a particular emphasis on resilience building. Resilience refers to the capacity to endure, recover, and flourish in the midst of adversities and existential anxieties. By having sufficient inner and external resources, adolescents can practice self-transcendence and transform life adversities into meaning, life goals, and healthy values that support their well-being [78]. In the TRY Gym project, psychoeducation was implemented not only to equip adolescents with coping strategies and skills to manage various troubles and threats but also to highlight internal factors such as individual traits and positive emotions, as well as external factors like supportive relationships and environments.

In addition, young people have been viewed as valuable resources to be developed under the framework of Positive Youth Development (PYD) [79,80,81]. Recognizing that adolescents possess considerable potential, it becomes evident that training them as health educators and leaders could yield long-term benefits in their knowledge, attitudes, leadership skills, and related behaviours [82]. By harnessing the strengths and capabilities of young people, this project aims to cultivate these attributes, fostering a generation of empowered and responsible individuals. Petrovskaya [83] suggested that experiential and service learning can shift adolescents’ values from self-enhancement to self-transcendence, helping them identify meaning, life goals, and healthy values with commitment, creativity, and prosocial behaviours. This shift is beneficial to their mental well-being. Therefore, the TRY Gym project actively involved youth in programme development and decision-making processes at school. Transformative practices through co-creation activities are included to support young people in exploring meaning, life goals, and healthy values through commitment, creativity, and prosocial behaviours. This approach not only enhanced the educational experience but also empowered students by giving them a voice and a role in shaping their personal development.

Lastly, the recovery concept and human library were also salient features of the TRY Gym training, aimed at reducing the stigma of mental illness. This interpersonal approach provides opportunities for people to meet individuals associated with the stigmatized group in a safe setting [84]. This aligns with Allport’s Intergroup Contact Theory [85], which proposes that supportive and collaborative contact between different social groups can reduce prejudice, with research suggesting a more significant effect than educational approaches [86]. By sharing first-hand accounts, these recoverees dispel preconceptions and give trainees a greater understanding of emotional and mental problems. This aligns with findings from Graziano et al. [76], where empathy, moderated by emotional self-efficacy, fosters inclusive interactions; in TRY Gym, such contact not only reduces stigma but also builds participants’ emotional regulation skills, enhancing their ability to transform adversities into growth opportunities. Through direct engagement, trainees foster empathy and knowledge, enabling them to see beyond stereotypes and appreciate the compassion and resilience of those dealing with mental health difficulties. Consequently, participants are more likely to develop empathy and reduce discriminatory actions, contributing to a more welcoming and supportive atmosphere for individuals with mental health issues.

### 1.5. Project Objectives and Research Hypothesis

The proposed programme aims to establish a bottom-up, proactive, co-creative, and positive youth approach to improve the psychosocial well-being of adolescents in Hong Kong, thereby addressing the concerning mental health issues among adolescents and the high student suicide rate. The programme’s main objectives include the following:

- To train a group of secondary students aged between 15 and 18 to be the youth mentors (trainees) with coping strategies, self-compassion, counselling, and empathic listening skills to increase their awareness of their own resilience and emotional competence. Trainees are expected to hang out with their peers/mentees with these skills.
- To construct positive relationships and a sense of belonging among adolescents. The programme will enhance the strengths of trainees to co-create timely, relevant, youth-driven online and/or onsite multimedia mental health educational projects for their peers/mentees in schools (aged 12–18) and people in the communities.
- To create a stigma-free and strengths-building platform to increase mental health literacy amongst trainees and their peers/mentees through online and onsite multimedia mental health educational projects.
- To develop the capability of trainees to identify their strengths and utilize support and resources in the community to build reciprocal support networks among trainees, their mentees, and the community.
- To promote positive youth development and the mental well-being of trainees and participating adolescents.

By completing the five objectives, three hypothesis sets were developed:

**H1:**  *Participants who completed the TRY Gym programme demonstrated significant improvements in developmental psychosocial competencies (emotional, social, motivational, cognitive, and self-competence) compared to their baseline levels before the programme.*

**H2:**  *Participants who completed the TRY Gym programme exhibited reduced levels of misconceptions and negative attitudes toward mental disorders compared to their baseline levels before the programme.*

**H3:**  *Participants who completed the TRY Gym programme showed improvement in mental well-being relative to their baseline levels before the programme.*

## 2. Materials and Methods

### 2.1. Participants and Recruitment

The target participants of this project were upper-form (Forms 3 to 5) secondary school students, while the lower-form students served as the beneficiaries of the youth-driven activities. The study employs specific inclusion and exclusion criteria to ensure participant suitability for the three-phase mental health programme. Inclusion criteria require participants to have an interest in mental health and its promotion, be willing to commit to the entire duration of the project spanning the academic year, and be able and willing to serve as a youth mentor and possess the ability to communicate effectively in either Cantonese or English. Exclusion criteria include individuals with suicidal ideation, those diagnosed with a mental illness, or those unable to sustain attention for at least 30 min. These criteria ensure that participants are well-suited to engage in the training workshop, self-practice phase, and implementation phase, while Form 6 students are excluded in the recruitment, as they typically begin study leave from mid-February to early March and they are intensively preparing for the upcoming DSE examinations. The recruitment of participants for the TRY Gym project was conducted in collaboration with four social service organizations in Hong Kong. These organizations facilitated the recruitment process through school social workers in eight secondary schools. The primary recruitment method was through referrals from teachers and social workers who nominated students they believed would benefit from the programme. Additionally, there was an element of open recruitment within the schools, where the programme was promoted to students through school-based advertisements. This dual approach integrating both referral and school-based advertisement ensured a comprehensive and targeted recruitment strategy for the project.

In the TRY Gym project, 104 students were recruited as trainees. Twenty-four trainees were excluded in the evaluation due to three situations: (1) lack of consent for data collection (*n* = 2); (2) incomplete data provision (*n* = 10); (3) withdraw from programme or study (*n* = 12). Consequently, 80 trainees were included in the study evaluation, with 19 participating in the interview section.

### 2.2. Programme Outline

To equip trainees with knowledge and skills to create their mental health promotion events, trainees underwent three stages: (1) the training stage, (2) the self-practice stage, and (3) the implementation phase. In the training stage, social work assistants from the four agencies conducted 8 sessions in each school, comprising talks, workshops, experiential activities, and human libraries. Participants were taught to identify and understand mental health issues, reduce stigma, build resilience, and practice self-compassion. They were also trained to develop empathy, discover personal strengths, explore community resources, and design and promote mental health programmes using social media. The details of each session can be found in Table 1. Manual and session plans were provided to social workers before the training stage. They also received trainings and instructions before the training stage. Also, fidelity sheets were provided to the workers. Social workers can rely on fidelity sheets to ensure they cover all the bullet points of session plans. Apart from materials, team members also observed sessions on-site from time to time. These methods can minimize the effect of social worker assistance on the training effects.

After the training stage, trainees went onto the self-practice stage, where they applied the skills and strategies learned independently in their daily lives, with materials provided to assist their implementation. During this stage, trainees were also required to discuss with social workers and school staff to plan and prepare their mental health promotion events. Only 1–2 school-based social workers (depending on the school’s specific circumstances) would participate in discussions with students about organizing activities and accompany them in the process, unless the scale of the activity required teacher assistance. Additionally, the school required review of the activities organized by students, for reasons including but not limited to student safety, venue availability, and manpower arrangements. Finally, in the implementation phase, trainees utilized the knowledge gained to organize their mental health promotion events within their schools or communities.

### 2.3. Measures

The TRY Gym programme was evaluated using an embedded mixed-methods approach that includes both quantitative self-reported surveys and qualitative one-on-one semi-structured interviews. To evaluate the effectiveness of the programme, participants completed the same set of psychological scales at three intervals: at baseline, post-training, and post-implementation. The survey used in this project included basic demographic information, such as age, gender, location of school, and secondary form, as well as twelve validated psychological scales described below. The psychological scales included validated measures of resilience, bonding, emotional, social, cognitive, and behavioural competence, self-efficacy, self-determination, and positive identity [87]. Additional scales measured stigma towards mental illness [88], self-compassion for youth [89], mental well-being [90], strength use and current knowledge scale [91], and Mental Help-Seeking Attitudes Scale [92]. The subsequent two questionnaires were similar to the baseline set, with the addition of an opinion section in the post-training and post-implementation phase questionnaires. The opinion section included five questions regarding perceived growth in understanding and awareness of mental health, the importance of recognizing and addressing mental health issues both personally and within the community, as well as emotional support, awareness, and knowledge of mental aid resources gained through the programme.

#### 2.3.1. Assessment of Positive Youth Development

To assess positive youth development, this study utilized a modified version of the Chinese Positive Youth Development Scale (CPYDS) [93], which has been validated for use with Hong Kong adolescents in prior research conducted in Hong Kong [87,93,94,95]. Detailed descriptions, items, and psychometric properties of the scale are available in a previous study [87]. The TRY Gym programme targets emotional, cognitive, behavioural, and social competence, as well as personal resilience, self-efficacy, and bonding, to enable young people to identify their strengths and support their growth, learning, and transformation. Accordingly, specific subscales of the CPYDS were selected: resilience (6 items), emotional competence (6 items), social competence (6 items), cognitive competence (6 items), self-efficacy (7 items), and self-determination (referred to as a sense of mastery in this study, 4 items). All selected subscales are measured on a 6-point Likert scale and have demonstrated high reliability in studies conducted in Hong Kong, with alpha coefficients of 0.77 or above [94,95]. These studies confirmed the scale’s cultural relevance and psychometric robustness for Hong Kong youth. In the present study, reliability tests yielded results consistent with previous findings: social competence (α = 0.881), cognitive competence (α = 0.852), resilience (α = 0.855), and sense of mastery (α = 0.809) subscales demonstrated good reliability, while emotional competence (α = 0.780) and self-efficacy (α = 0.737) subscales showed good to acceptable internal consistency. All referenced studies [87,93,94,95] were conducted in Hong Kong and reported satisfactory internal consistency, supporting the suitability and reliability of the CPYDS for this population.

#### 2.3.2. The Revised Motivation Scale, the Student Opinion Scale (SOS)

The revised Motivation Scale, and the Student Opinion Scale (SOS) [96] were built on the first version of the motivation scale [97]; they added two items and revised the wordings of other items. The motivation scale was designed to measure an individual’s motivation on tests. It had a high reliability, with over 0.80 consistently in the alpha coefficient when tested among 15,000 students [98]. The SOS used a 5-point Likert scale consisting of 10 items. The setting of the scale shifted from tests to the programme designed for this study. Although the alpha value is generally considered as acceptable at 0.8 for cognitive tests, and 0.7 for ability tests, below 0.7 can also be expected for psychological constructs values [99]; the reliability of this motivational competence scale of 0.681 could be considered as marginal at best.

Previous studies provide indirect support for the cultural and linguistic appropriateness of the Student Opinion Scale (SOS) in Hong Kong contexts, particularly through its prior application with local student populations and evidence of adaptations in scale development. Specifically, some previous studies [100,101] successfully employed the SOS among Hong Kong-based Chinese students, demonstrating its feasibility in a culturally similar setting, and their Cronbach’s alpha values were 0.71 [101] and 0.82 [100], respectively. Although these studies recruited undergraduates who may not exclusively be adolescents, the participants were drawn from the same geographic and cultural milieu as our high school student sample, including shared linguistic influences from Cantonese and English in Hong Kong’s bilingual education system. This prior usage implies a degree of cultural relevance, as the scale was administered without reported issues in comprehension or applicability, and it yielded interpretable results in these local cohorts.

#### 2.3.3. The Perceived Devaluation-Discrimination Scale

The Perceived Devaluation-Discrimination Scale [88] was employed to assess participants’ perceptions of stigma toward current or former psychiatric patients. This 12-item, 5-point Likert scale measures the extent of agreement with statements reflecting societal devaluation of psychiatric patients (e.g., perceiving them as failures, less intelligent, or having opinions not taken seriously) and discrimination in social relationships. The scale has demonstrated robust psychometric properties, with a global internal consistency of α = 0.78 [102]. In the present study, the internal consistency was slightly higher at α = 0.805, indicating good reliability within our sample of Hong Kong adolescents.

To ensure the scale’s appropriateness for the Hong Kong adolescent population, we utilized the Chinese version of the Perceived Devaluation-Discrimination Scale, which has been previously validated in Hong Kong with a general population aged 18 and older across three population-based surveys between 2009 and 2018 (*n* = 3548) [103]. This prior research reported an internal consistency of α = 0.76, confirming the scale’s reliability in the local context.

#### 2.3.4. The Warwick–Edinburgh Mental Well-Being Scale (WEMWBS)

The Warwick–Edinburgh Mental Well-being Scale (WEMWBS) [90] is a validated instrument designed to monitor mental well-being at a population level and evaluate mental health promotion initiatives. It consists of 7 positively worded items assessing various aspects of positive mental health, rated on a 5-point Likert scale. The scale has demonstrated high reliability in prior studies, with Cronbach’s alpha of 0.89 in a UK student sample and 0.91 in a UK population sample [90]. In the present study, the WEMWBS exhibited good reliability among Hong Kong adolescents, with a Cronbach’s alpha of 0.816.

To ensure cultural and linguistic appropriateness for the Hong Kong adolescent population, the Chinese version of the Warwick–Edinburgh Mental Well-being Scale (WEMWBS) was employed. The translation process followed a rigorous forward-step, backward-step, and pretest-step method, as outlined in a previous study [104]. The Chinese WEMWBS demonstrated moderate to good reliability in the general Hong Kong population (Intraclass Correlation Coefficient = 0.70, 95% CI 0.55 to 0.80, *p* < 0.001) [104], consistent with an earlier Chinese version validated among hospitalized patients with mental illness in Hong Kong (Cronbach’s alpha = 0.89) [105]. These validation studies confirm the reliability and validity of the Chinese WEMWBS for use in Hong Kong populations, including adolescents, supporting its appropriateness for the current study.

#### 2.3.5. Self-Compassion Scale for Youth

The Self-Compassion Scale for Youth (SCS-Y) was developed to measure self-compassion in adolescents, comprising 17 items across six subscales: self-kindness, mindfulness, common humanity, self-judgment, isolation (three items each), and over-identification (two items), rated on a 5-point Likert scale [89]. Previous research reported acceptable reliability for each subscale, with Cronbach’s alpha exceeding 0.70 [89]. In the present study, the SCS-Y demonstrated good reliability among Hong Kong adolescents, with a Cronbach’s alpha of 0.755, consistent with prior findings.

Regarding cultural and linguistic appropriateness for the Hong Kong adolescent population, the SCS-Y was validated in a study involving 1777 Chinese adolescents (including those from Hong Kong) aged 12–18 years [106]. This research employed a Multiple Indicator Multiple Cause (MIMIC) model to confirm the psychometric properties of the SCS-Y, reporting a Cronbach’s alpha of 0.76 for the overall scale, indicating good reliability. The study also investigated differential item functioning across Chinese, Hong Kong, and UK adolescents, confirming the scale’s cultural appropriateness and measurement invariance for use with Hong Kong adolescents. These findings support the reliability and validity of the SCS-Y for the current study’s adolescent sample in Hong Kong.

#### 2.3.6. Strengths Use and Current Knowledge Scale

Jarden [91] adapted a 7-point Likert scale—the Strength Use and Current Knowledge scale, a 14 to 10 item version of the scale developed by Govindji and others [106]. It was developed to measure strengths knowledge and strength use, which referred to people’s awareness and recognition of their strengths and how much people use their strengths in a variety of settings [107]. The scale is a reliable measure across cultures, with Cronbach’s alpha coefficients ranging from 0.95 [107] to 0.97 [108]. The result of Cronbach’s alpha coefficients in this study was α = 0.871, which was slightly lower than the previous study but still indicated good reliability.

To ensure cultural and linguistic appropriateness for the Hong Kong adolescent population, the Chinese version of the Strengths Use and Current Knowledge Scale was adopted from a cross-cultural study on satisfaction with life involving participants aged 16 or above [109]. The scale was translated into multiple languages, including Chinese, with internal consistency coefficients for the overall scale ranging from 0.86 to 0.92 across all languages, confirming its reliability and validity for use in Chinese populations.

#### 2.3.7. Mental Help-Seeking Attitudes Scale (MHSAS)

The Mental Help-Seeking Attitudes Scale is a 9-item, 7-point Likert scale developed to measure one’s evaluation of their seeking help from a mental health professional [108]. In the present study, the MHSAS showed comparable reliability among Hong Kong adolescents, with a Cronbach’s alpha of 0.877.

To ensure cultural and linguistic appropriateness for the Hong Kong adolescent population, the Chinese version of the MHSAS was adopted, as validated by Xu et al. [110]. The translation process followed a rigorous standard procedure involving two forward translations, two backward translations, and five cognitive interviews to confirm linguistic and cultural suitability. The Chinese MHSAS was tested with 1008 participants aged 16–30 years in Guangzhou, China, yielding a Cronbach’s alpha of 0.88, indicating strong reliability. These findings confirm the reliability and validity of the Chinese MHSAS for use in Chinese-speaking populations, supporting its appropriateness for the current study.

#### 2.3.8. Qualitative Interviews

Based on the theoretical framework and objectives of TRY Gym, a protocol was developed, which was used in the pilot TRY Gym. After the pilot, experts were invited to review and revise the guidelines. Before the interview, the protocol was also sent to schools and social workers for preview, and revised based on their advice. Then the protocol was implemented in this programme to conduct one on one pre- and post-interviews between the researcher and participants. The pre-interview protocol covered the reasons for joining the programme, and perspectives on mental health topics and agenda. Participants were asked about their mental health and that of the people around them. Questions regarding their understanding of mental health and self-assessment were included to establish a baseline for comparison with the post-interview. In the post-interview, questions were set to link back to the pre-interview’s answers. These questions addressed the issues and concerns expressed by trainees during the pre-interview, with responses used to determine whether those issues and concerns were addressed throughout the programme. The post-interview protocol also covered the knowledge and skills learnt from the programmes, changes in self-perspective and competencies, and school culture. Besides assessing personal changes and surroundings, a set of questions was included to gather trainees’ opinions and suggestions for the programme, which could be used for future project improvements and developments.

### 2.4. Procedures

**Figure 2:**
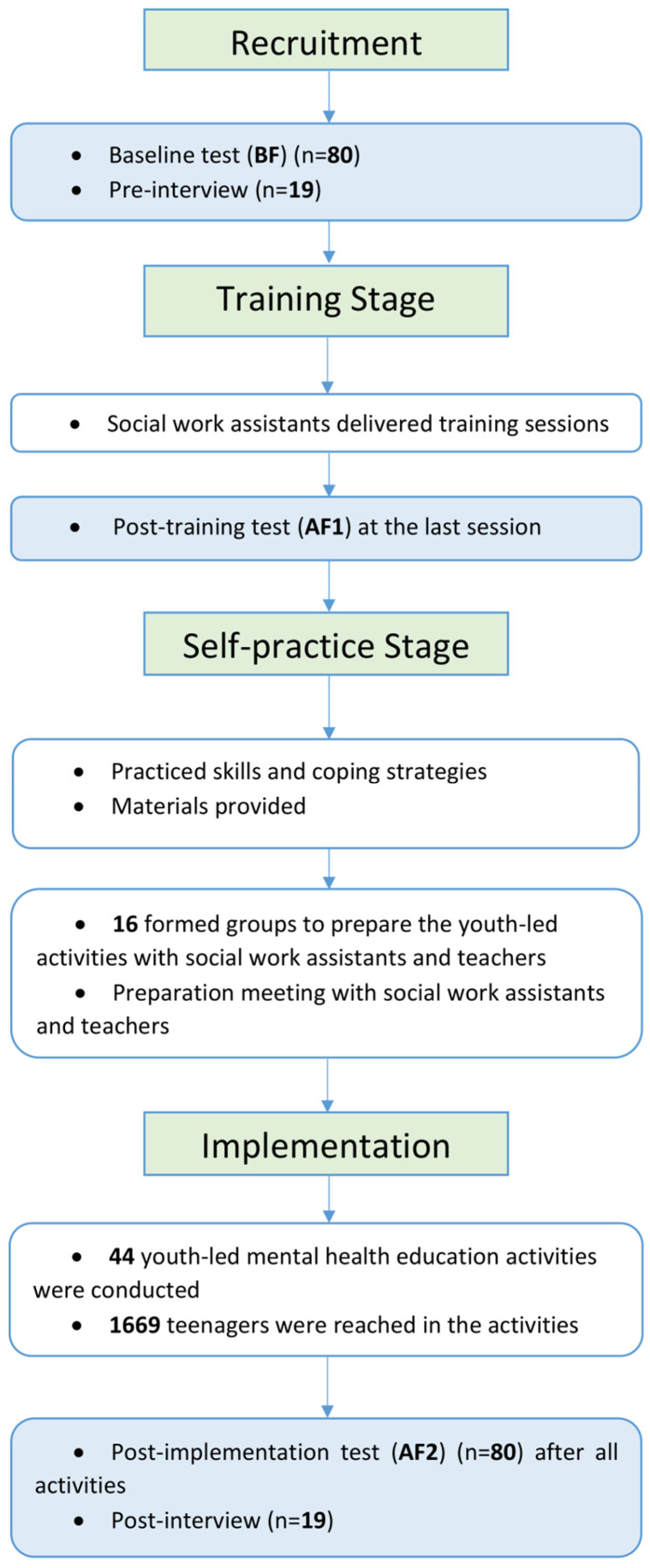
Flowchart of the programme.

Before the programme, participants and their parents were provided with an information leaflet and a consent form describing the project’s aim and outline, along with its research purpose and procedures. Upon providing informed consent, participants were given either a hard copy or a hyperlink to the baseline questionnaire, completed at or before the first session. Participants then attended the eight sessions of training and completed the survey for the second time to assess the changes. Subsequently, participants entered a four-week practice stage, during which they engaged in structured activities. In addition, 16 groups were formed, each tasked with designing and organizing at least three mental health-related events across six months, Figure 2.

During the implementation phase, participants organized a total of 44 activities aimed at promoting and disseminating mental health prevention information within their school, neighbouring schools, and communities. Upon completing the implementation phase, participants were administered the same post-training survey again. Those who had participated in the pre-interviews were interviewed again to evaluate their overall experiences and gains throughout the programme. During the interview, one registered social worker from the school or research team would present. The interview might involve sensitive topics, including but not limited to self-harming behaviours and suicides. So, social workers during the interview could be an ally for the interviewee and provide emotional support to them. The arrangement concerned the interviewees’ safety and health. Before the interview, interviewees were informed of the presence of a social worker and then offered an option of either a social worker from his/her school or from the research team. The option managed to reach a balance between familiarity and disclosure. Also, since Hong Kong is a tri-lingual society, participants could choose among Cantonese, Putonghua, and English based on their preferences. All interviews were audio-recorded with consent obtained from the participants.

### 2.5. Data Analysis

The calculation and analysis of changes in scores on psychological outcomes over time were performed by the IBM SPSS Statistics Package 26. Before analysing the quantitative data, mean substitution and coding were conducted to facilitate further analysis. Little’s Missing Completely at Random (MCAR) test confirmed that the missing data pattern is MCAR, with a missing data rate of 0.02%. Therefore, mean substitution is an acceptable method for replacing missing data. Boxplots of items with missing data were examined, and no outliers were identified.

The mean score of each item in the scale was calculated and substituted into the missing data value for that same variable to allow the utilization of collected data in an incomplete dataset. Besides that, considering the categorical data collected in the demographic sections (for example, gender, living districts, etc.), label encoding was used for assigning a unique integer value for each unique category.

A statistical power analysis was conducted to estimate sample size. A medium effect size of 0.25 for repeated measures Analysis of Variance (ANOVA) was adopted [111]. Additionally, a medium effect size of 0.30 for chi-square tests (goodness of fit and contingency) was used to ensure sufficient sample size for the Friedman test. With an alpha of 0.05 and power of 0.95, the estimated sample sizes required (using GPower 3.1.9.7) were approximately *n* = 43 for ANOVA and *n* = 31 for the chi-square tests in within-group comparisons.

Statistical analysis was then performed. A normality test was first used to examine data distribution. If all three phases of a scale were normally distributed, the repeated measures Analysis of Variance (ANOVA) was used to analyse the data [112]. If the data were not normally distributed, the Friedman test was a non-parametric repeated measures Analysis of Variance by ranks across the three stages. It analyses differences in distributions across three groups by ranking the data for each subject and comparing the sum of ranks across conditions using a chi-square statistic [113]. Dunn’s pairwise post hoc tests were used to analyse pairwise comparison among three phases for scales with a significant Friedman test result to explore the relationship among three phases. To calculate the effect size of the post hoc test of the Friedman test, the formula r = z/√N was applied [114].

This analysis aimed to determine whether there were significant changes in the measured variables over time, determining the effectiveness of the programme.

All interviews were recorded and then transcribed by Buzz 1.20, which is a software assisting offline audio transcription and translation, powered by OpenAI’s Whisper. Generated texts were modified and proofread by the team to ensure the accuracy of transcription.

Deductive thematic analysis was then implemented [115]. The themes were determined by the project outline highlighting the five core competences that our programme extracted from the PYD framework. Pre-test themes were applied in the coding. Two coders were involved, and both coders were also interviewers. So, when coding the transcripts, at least one coder had seen and interviewed interviewees on-site or online. This arrangement can ensure that some information in the interviews such as tones and body gestures would not be missed during the transcription.

To ensure consistency and reliability of the coding process, three out of nineteen interview transcripts were selected to implement an intercoder reliability test. Two coders coded these transcripts using the pre-test themes. Then the Cohen’s Kappa was calculated to measure the level of agreement between two coders. And the Kappa value was 0.71.

Discrepancies were discussed and resolved through consensus meetings, where adjustments to the coding framework were made to enhance clarity and consistency. This process contributed to the overall rigor and trustworthiness of our thematic analysis. This method allowed the identification of common patterns and insights related to the programme’s impact on participants, complementing the quantitative findings and providing a comprehensive evaluation of the programme’s effectiveness.

### 2.6. Ethical Considerations

Ethical approval was obtained from the Human Research Ethics Committee of the higher education institutions. Potential ethical concerns were addressed prior to the study, particularly concerning the self-discovery journey in the training, which might have aroused discomfort among participants, especially since most of them were under 18 years of age. To mitigate these concerns, the programme objectives, potential harms, benefits, voluntary participation in the evaluation, and the right to withdraw were clearly explained to the participants. Informed consent was obtained from both participants and their parents or guardians for further analysis of the project.

## 3. Results

### 3.1. Demographics Information

The evaluation study sample consisted of 80 participants with a mean age of 16.05 years (SD = 0.95), ranging from 14 to 19 years. The gender distribution was 46.3% male (*n* = 37) and 53.8% female (*n* = 43). Participants attended schools from various locations: 23.8% were from Hong Kong Island (*n* = 19), 36.3% from Kowloon (*n* = 29), and 40.0% from the New Territories (*n* = 32). The participants were drawn from eight secondary schools that received an eight-session programme from different agencies.

Training workshops were conducted at the eight secondary schools with a total of 104 secondary student trainees aged from 14 to 19; however, twelve of them did not complete the entire programme. Consequently, 80 of the 92 trainees were included in the data analysis procedure, with two excluded due to being denied consent for data collection and 10 excluded due to incomplete data, resulting in an 86.9% response rate. In terms of academic level, 13.8% were Form 3 students (*n* = 11), 55.0% were Form 4 students (*n* = 44), and 31.3% were Form 5 students (*n* = 25). Regarding housing, 30.4% resided in public housing (*n* = 24), 15.2% in Home Ownership Scheme units (*n* = 12), 28.1% in private housing (*n* = 38), and 7.5% in other types of housing (*n* = 6). Demographic information is summarized in Table 2.

### 3.2. Quantitative Results

According to the normality test (see Table 3), all the variables were not normally distributed across three time points.

**Figure 3:**
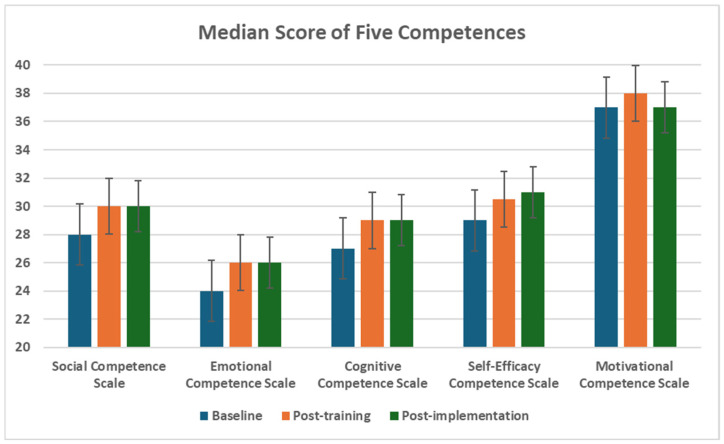
Median score of five competences. This figure tracks five competence scales across three phases. Scores generally improved post-training, with stability or minor gains post-implementation: Social, Emotional, and Cognitive showed consistent positive shifts; Self-Efficacy saw steady progress; Motivational had a slight uptick then dip. Overall, training yielded positive changes in social, emotional, and cognitive areas.

**Figure 4:**
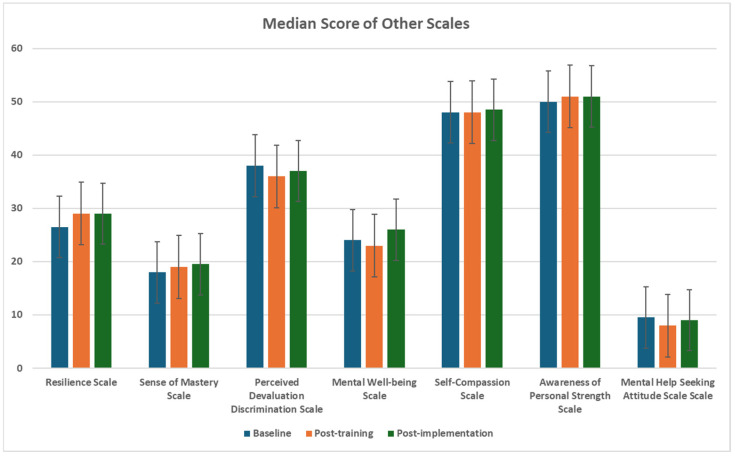
Median score of other scales. The range of the Mental Help-Seeking Attitudes Scale is from −27 to 27. This figure monitors seven well-being-related scales. Trends show mixed but mostly upward movement: Resilience and Mastery improved steadily; Devaluation/Discrimination decreased initially then rose slightly; Mental Well-being dipped then rebounded; Self-Compassion and Personal Strength were stable with slight gains; Help-Seeking Attitude fluctuated. Implementation phase reinforced gains in resilience and well-being.

Hence, the non-parametric statistical test—the Friedman test—and Dunn’s pairwise post hoc tests were used to analyse differences in distributions by ranks for all scales. Table 4 shows the scores of all scales at baseline, post-training, and post-implementation phases, results of the Friedman test and its post hoc test, and the effect size of the post hoc test. Moreover, the Benjamini–Hochberg (BH) adjusted *p*-value controls the False Discovery Rate (FDR), the expected proportion of false positives among all significant results. It addresses the problem of multiple comparison, where testing many hypotheses at the same time increases the chance of false positives (Type I error) [116]. It was applied to adjust the *p*-value of the Friedman test. Besides, the Dunn’s pairwise post hoc tests provided the adjusted *p*-value by Bonferroni correction for the post hoc test of the Friedman test. Bonferroni correction is a common way to address Type I error by dividing the *p*-value by the total number of performed tests [116], Figure 3 and Figure 4.

#### 3.2.1. Survey Results

##### Social Competence

The TRY Gym programme significantly enhanced social competence, as evidenced by the Friedman test results (χ^2^(2) = 20.216, *p* < 0.001). Post hoc analyses confirmed significant increases in median scores from 28 at baseline to 30 at both post-training (*p* = 0.001, r = 0.41) and implementation phases (MDN = 30; *p* = 0.002, r = 0.38). These findings indicate the programme effectively improved participants’ social skills, fully supporting H1 for social competence.

##### Cognitive Competence

Cognitive competence showed significant improvement following the TRY Gym programme (χ^2^(2) = 13.251, *p* = 0.003). Median scores increased from 27 at baseline to 29 at post-training (*p* = 0.009, r = 0.33) and implementation (MDN = 29; *p* = 0.009, r = 0.33), reflecting enhanced cognitive abilities. This consistent gain across phases strongly supports H1 for cognitive competence.

##### Emotional Competence

Emotional competence significantly improved (χ^2^(2) = 9.017, *p* = 0.022), with median scores rising from 24 at baseline to 26 at implementation (*p* = 0.024, r = 0.30), suggesting a modest positive effect on emotional regulation and supporting H1. Resilience also showed significant gains (χ^2^(2) = 16.050, *p* = 0.001), with medians increasing from 26.5 at baseline to 29 at post-training (*p* = 0.001, r = 0.41) and implementation (MDN = 29; *p* = 0.024, r = 0.30), indicating an enhanced ability to cope with challenges and supporting H1. However, self-compassion exhibited a nonsignificant increase (χ^2^(2) = 1.199, *p* = 0.732), with medians stable at 48 from baseline to post-training and slightly rising to 48.5 at implementation, partially supporting H1 and suggesting a need for further investigation.

##### Self-Efficacy Competence

Self-efficacy competence showed a nonsignificant but directional trend toward improvement (χ^2^(2) = 5.062, *p* = 0.136), with median scores increasing from 29 at baseline to 30.5 at post-training and 31 at implementation, indicating a potential boost in confidence and partially supporting H1. Sense of mastery significantly improved (χ^2^(2) = 16.572, *p* = 0.002), with medians rising from 18 at baseline to 19 at post-training (*p* = 0.015, r = 0.31) and 19.5 at implementation (*p* = 0.001, r = 0.40), reflecting enhanced feelings of control and supporting H1. Awareness of personal strengths showed a nonsignificant trend (χ^2^(2) = 0.164, *p* = 0.921), with medians increasing from 50 at baseline to 51 at post-training and implementation, partially supporting H1.

##### Motivational Competence

Motivational competence showed no significant change (χ^2^(2) = 0.433, *p* = 0.967), with median scores fluctuating from 37 at baseline to 38 at post-training and back to 37 at implementation. This lack of consistent improvement indicates the programme did not effectively enhance motivational competence, failing to support H1.

##### Misconceptions and Negative Attitudes Toward Mental Disorders

The programme did not significantly reduce misconceptions or negative attitudes toward mental disorders, failing to support H2. For Positive Attitudes Toward Seeking Services (MHSAS), the Friedman test showed no significant changes (χ^2^(2) = 0.351, *p* = 0.915), with medians slightly decreasing from 9.5 at baseline to 8 at post-training and rising to 9 at implementation, indicating minimal net change. Similarly, Perceived Devaluation-Discrimination showed nonsignificant changes (χ^2^(2) = 4.252, *p* = 0.179), with medians decreasing from 38 at baseline to 36 at post-training but increasing to 37 at implementation, suggesting limited effectiveness in reducing stigmatizing perceptions.

##### Well-Being

The TRY Gym programme significantly improved mental well-being (χ^2^(2) = 14.676, *p* = 0.002), with median scores increasing from 24 at baseline to 26 at implementation (*p* = 0.002, r = 0.39) and from 23 at post-training to 26 at implementation (MDN = 26; *p* = 0.022, r = 0.30). These results reflect a positive impact on overall mental health, strongly supporting H3.

##### Opinions About TRY Gym

Participants’ opinions regarding the training and implementation phase were positive, with all mean scores exceeding 4 on a 5-point Likert scale. Moreover, the trend for all items showed an upward trajectory. The descriptive statistical results for the opinion scale are displayed in Table 5, indicating that participants felt the training and implementation phase effectively enhanced their understanding and awareness of mental health.

### 3.3. Group Interview Results

Two or three trainees were randomly selected from each school to participate in the focus group interviews, with 19 students interviewed in total. The qualitative interviews aimed to explore participants’ experiences in the programme across various aspects, focusing on the five competencies: emotional, social, motivational, cognitive, and self.

As mentioned in the data analysis section, to ensure consistency and reliability of the coding process, three out of nineteen interview transcripts were selected to implement an intercoder reliability test. Two coders coded these transcripts using the pre-test themes. Kappa was calculated to measure the level of agreement between two coders, and the Kappa value was 0.71, indicating substantial agreement between two coders, suggesting that the coding was applied consistently. The framework and coded sections were revisited and reviewed. Discrepancies were discussed and resolved through consensus meeting, and the framework was refined based on the discussion.

#### 3.3.1. Emotional Competence

Emotional competence emerged as the most frequently mentioned skill in the interviews. Participants highlighted how TRY Gym helped them acquire emotional regulation techniques, reporting they lost their temper less frequently. They also knew how to de-escalate tense situations, such as avoiding bringing up past mistakes during conflicts with their parents. Through deep breathing and mindfulness practices, they learned to remain calm and composed, such as one trainee mentioned,

*I was easy to lose temper, but now I figure out how to control it.*

(Trainee no. 3-2-4. Male, 17 y/o, family income: HKD 10,001–20,000, housing type: HOS, Cantonese speaker.)

*It has made my mood swings smaller than before, and my emotions are more stable.*

(Trainee no. 4-2-9. Female, 15 y/o, family income: not sure, housing type: private, Cantonese speaker.)

Interactions with friends and colleagues and more harmonious relationships led to improved emotional competence. This ability to manage emotions was a significant benefit of TRY Gym.

Self-compassion, an important element of emotional competence, was also highlighted. Trainees described self-compassion as understanding how to care for themselves instead of engaging in excessive self-criticism. For instance, they previously exaggerated the significance of lower marks on mock tests or minor quizzes, believing it indicated total failure. After participating in TRY Gym, they recognized those tests and quizzes are just practice and learned to adjust their mindsets and be kinder to themselves.

*Except for being judgmental, I also need to care for myself. I think I learnt how to change my mentality on this.*

(Trainee no. 4-1-3. Female, 15 y/o, family income: not sure, housing type: private, Cantonese speaker.)

*I think I am much better in mental health than before. I don’t overthink the negative stuff. Before if I got a bad score in a quiz, I would feel so screwed. Now I feel more confident.*

(Trainee no. 3-2-4. Male, 17 y/o, family income: HKD 10,001–20,000, housing type: HOS, Cantonese speaker.)

This shift in perspective reduced harsh self-judgment and allowed them to approach challenges with a more balanced and forgiving mindset, improving emotional well-being, resilience, and motivation.

#### 3.3.2. Social Competence

Social competence involves bonding with peers and adopting positive social roles. Empathy was notably observed in the post-test interviews. All trainees, being school students, often faced challenges related to gossip, which lead to emotional struggles. After participating in the TRY Gym programme, participants expressed greater mindfulness toward those in distress, emphasizing the importance of supporting peers to ensure they feel less alone, as some trainees highlighted.

*Because, you know, like high school gossip, some people have difficulties in their mental health. So, yeah, whenever I encounter those people that I’ve heard that they’re struggling mentally, I just become more careful and act normal.*

(Trainee no. 2-2-15. Female, 15 y/o, family income: not sure, housing type: private, English speaker.)

*However, I pay attention to other people’s emotions. I have noticed that if someone around me is unhappy, I will quickly try to understand or meet them face-to-face. In the past, I would constantly say comforting words to them and try to instil my own ideas. But now I have learned to support them and be there for them with genuine companionship.*

(Trainee no. 3-2-2. Male, 17 y/o, family income: HKD 10,001–20,000, housing type: monthly rental, Cantonese speaker.)

They described how the programme helped them understand the impact of their actions and words, leading to more considerate interactions. This newfound empathy strengthened friendships and fostered a more inclusive school environment, enhancing their social competence and contributing to a positive community culture. 

Social competence also encompasses improved social skills. Some trainees mentioned difficulties in building connections before the programme. After TRY Gym, many reported feeling more confident and less anxious in social situations, enabling them to engage in small talk and make friends.

*I seldom talk to strangers. But after this programme, for example, when I was at the food court, and I shared a table with a stranger. If I felt he wanted to talk to me, then I could start conversations, just being more extroverted.*

(Trainee no. 1-2-8. Male, 16 y/o, family income: not sure, housing type: public, Cantonese speaker.)

*I am in the higher form, so I am older than the other participants in the events. After getting to know them, I am more willing to meet new people and make friends. I am open to chatting with them and expanding my own social circle.*

(Trainee no. 1-2-4. Male, 16 y/o, family income: HKD 30,001–40,000, housing type: private, Cantonese speaker.)

They also noted they learned more about other trainees during the implementation phase, building a sense of belonging and connection. The programme’s focus on social competence provided tools for better social interactions, leading to increased self-assurance and communication skills. This improved ability to connect with others enhanced personal relationships and contributed to a supportive community atmosphere. 

#### 3.3.3. Motivational Competence

During activities, interviewees demonstrated a high level of intrinsic passion, dedicating their time and energy to the programme for their growth rather than for a certificate. Despite TRY Gym providing no incentives beyond certificates, trainees still regarded the project as an important part of their school life, indicating deep-seated intrinsic motivation. Participants eagerly participated in activities and workshops, driven by a genuine desire to improve and learn new skills. This passion was evident in their enthusiastic approach to tasks, setting personal goals, and taking pride in their progress. The programme’s focus on personal growth nurtured this motivational competence, encouraging trainees to find joy and satisfaction in learning. Their actions and attitudes during TRY Gym demonstrated a strong internal drive, contributing to individual development and a richer collective experience. 

Persistence was also evident in youth-led activities. Trainees expressed that while the time devoted to these activities was long, it was worthwhile. They shared stories of how extended preparation helped develop better organizational and time-management skills, emphasizing teamwork and effective communication.

*When we were preparing activities, what gave me the deepest impression was that we needed to design games and props for the games. And we Care Ambassadors also prepared and packed more than 20 gifts. Most of us had been preparing for hours, and that’s a long time.*

(Trainee no. 1-2-8. Male, 16 y/o, family income: not sure, housing type: public, Cantonese speaker.)

*I learned about team spirit and how to express my emotions. In the past, when forced to do something, I would choose to remain silent, but now I actively express my thoughts.*

(Trainee no. 4-2-2, Female, 14 y/o, family income: not sure, housing type: HOS, Cantonese speaker.)

The programme’s structure, emphasizing youth leadership and initiative, fostered this persistence, creating an environment where trainees felt responsible for their efforts’ outcomes. This persistence enhanced their problem-solving abilities and instilled a sense of resilience that extended beyond the programme. The persistence demonstrated by trainees in managing youth-led activities underscored their ability to commit to long-term projects despite challenges, making the experience a valuable part of their school life.

#### 3.3.4. Cognitive Competence

Cognitive competence was reflected in the skills participants learned through the TRY Gym programme. Interviewees reported acquiring micro-counselling skills, making communicating with older individuals easier and avoiding conflicts. These skills enabled them to navigate conversations with greater ease and understanding, fostering more harmonious interactions. Additionally, some interviewees noted they became better equipped to talk with peers experiencing emotional issues, learning to listen actively, offer support, and de-escalate tense situations, as some trainees elaborated.

*So, I feel like that was also very meaningful and like ways to talk to people and like counselling and like yeah, just talking to people with. It’s a little bit more sensitive in general.*

(Trainee no. 2-2-17. Female, 16 y/o, family income: not sure, housing type: private, English speaker.)

*I have now learned how to better communicate with others, understand their feelings, and practice empathy. I used to be someone who was centred on my own feelings. Now, when I talk or argue with others, I try to put myself in their shoes and consider their thoughts or feelings.*

(Trainee no. 1-1-1. Female, 15 y/o, family income: not sure, housing type: private, Putonghua speaker.)

Beyond these communication skills, participants also developed personal coping strategies, though they did not specifically label them as such. They learned to manage overthinking and relax when facing academic and social stress. Despite the diversity of the eight participant schools in TRY Gym, all interviewees mentioned experiencing academic and social stress in the pre-interviews. In the post-interviews, most expressed that they had obtained personal coping strategies at different levels, helping them deal with these pressures more effectively.

*I learnt to do the meditation. Now, when I feel stressed, I will meditate to relieve stress. I think the skill can help me a lot.*

(Trainee no. 1-1-11. Female, 15 y/o, family income: HKD 40,001–50,000, housing type: public, Cantonese speaker.)

*I felt like before the programme, I was often suspicious and worried, sometimes being anxious over things that didn’t need concern, or just experiencing fear for no reason. But after participating in the programme I realized that there are many ways to help relieve stress. I also learned that many things aren’t as tense or difficult as I imagined, and I don’t need to be so fixated on them. Some issues can be solved quite easily. Perhaps by actively seeking help from friends, reaching out to those around you, or doing something very simple.*

(Trainee no. 2-1-4. Male, 15 y/o, family income: not sure, housing type: private, Cantonese speaker.)

#### 3.3.5. Self-Competence

In terms of self-competence, interviewees demonstrated a better understanding of their strengths. During sessions with social workers, they identified their abilities through guided activities. Although initially challenging, participants ultimately recognized their unique qualities and capabilities, finding the process enlightening and empowering.

*And then there were things like, what was really like impactful towards me in that session was that writing my weaknesses was really easy, but then writing my strengths, I couldn’t really think of anything. So, I mean, reflect on myself a lot.*

(Trainee no. 2-2-15. Female, 15 y/o, family income: not sure, housing type: private, English speaker.)

*I feel that my perspective on myself has changed. I used to think that others were living well and that my own existence seemed useless. Now I feel that my sense of self-worth has improved.*

(Trainee no. 4-2-2. Female, 14 y/o, family income: not sure, housing type: HOS, Cantonese speaker.)

By discovering and acknowledging their strengths, participants felt a boost in self-confidence and self-awareness, allowing them to leverage their strengths in various aspects of their lives, from academics to social interactions, enhancing their overall personal development. 

Another aspect of self-competence highlighted was the development of a positive identity. Some interviewees reported feeling more confident and adopting a more positive outlook on life since participating in TRY Gym.

*I feel my attitude towards life has been changed. I am more optimistic and complain less than before.*

(Trainee no. 4-2-2. Female, 14 y/o, family income: not sure, housing type: HOS, Cantonese speaker.)

*I feel that I have become more optimistic. I was originally quite a pessimistic person, so I feel more optimistic now. In the past, when I was more pessimistic and didn’t really believe in myself, I felt that things were pretty bad.*

(Trainee no. 3-2-3. Male, 16 y/o, family income: HKD 10,001–20,000, housing type: public, Cantonese speaker.)

They expressed a strengthened sense of self, suggesting they had developed more positive identities. Through activities and reflections, participants gained a better understanding of their values, interests, and aspirations, appreciating their unique qualities. This positive shift in self-perception empowered them to tackle challenges with greater confidence and resilience, making them more willing to take on leadership roles and pursue their goals.

#### 3.3.6. Unique Features of TRY Gym

During the analysis, apart from the five competencies, several common themes were repeatedly mentioned among interviewees. These were the most frequently highlighted elements of the programme that participants considered unique compared to other similar mental health activities. An additional subsection was included to elaborate on these elements.

##### Human Library Sharing from Persons in Mental Recovery

The human library sharing of the challenges and recovery stories was eye-opening for trainees. It was common that trainees believed that the human libraries helped them understand more about emotional/mental disorders, and lowered their stigmatization and potential discrimination against mental health.

##### Relaxing and Inclusive Space for Trainees

Most workshops and youth-led activities were held in schools during lunch breaks or after school hours. However, it was still crucial to maintain a stress-free atmosphere for trainees. During the workshop, trainees were encouraged to talk with each other, but not mandatorily. In most schools, trainees did not know all the trainees, so they practiced and developed more social skills. One trainee mentioned that he could talk to strangers more easily than before. Also, the workshops were relaxing, giving trainees a break from academic stress.

##### Hands-On Experiences of Organizing Youth-Led Activities

For most of the trainees, organizing a mental health activity was a new experience. So, they spent time both on-site and online discussing and preparing activities. On that day, they also needed to run the event. Social workers would guide them, but they needed to finish most of their duties independently, which is the essence of youth led. Through organizing youth-led activities, resilience, a sense of mastery, and awareness of personal strength were improved among trainees.

## 4. Discussion and Summary

The evaluation revealed significant improvements in participants’ social, emotional, cognitive, and self-competence, as well as in resilience and mental well-being, as evidenced by comparative scale scores at the post-implementation phase relative to baseline and post-training assessments. These findings align with the programme’s intended outcomes, as it was specifically designed to foster growth in these domains. While changes in mental health-seeking attitudes, awareness of personal strengths, and sense of mastery did not reach statistical significance, the observed trends were consistent with expectations, showing a progressive increase from baseline to post-training and post-implementation phases. These results support the programme’s broader aims of strengthening participants’ developmental psychosocial competence and personal growth, laying a solid foundation for improved mental well-being.

### 4.1. Programme Outcome

Throughout the course of the programme, the first goal of raising adolescents’ (trainees and non-participating adolescents) understanding and awareness of mental health issues was accomplished. In the first year of the project, 94 secondary students (aged 15–18) across eight schools were trained and became youth trainees through participating in eight sessions of TRY Gym Training Workshops. The promotion and support of positive youth development and adolescents’ mental health were achieved by conducting 44 youth-led activities, engaging 1669 teenagers in a range of student-led activities (e.g., mindfulness yoga, board games for relaxation, etc.) and through the programme’s digital outreach on social media, which increased its impact and created a setting that supported positive youth development and mental health among secondary students. Following the activities, teenagers felt quite satisfied with the experience (8.35 out of 10) and would recommend and urge others to participate (8.17 out of 10). Adolescents also indicated on the feedback form that they had better mental well-being (on average 3.36 out of 5) and increased knowledge and understanding of mental health issues (on average 3.42 out of 5). These findings implied that the TRY Gym programme had a broad impact on enhancing mental well-being, benefiting not only participants but also other adolescents at school.

### 4.2. Hypothesis Set 1: 5 Core Competencies

The 80 trainees included in the study, on average, demonstrated significant improvements in social, emotional, cognitive, and self-competence, when comparing the corresponding scale scores at the post-implementation phase with the scores at the baseline and post-training stage. These significant patterns partly agreed with H1, confirming the effectiveness of the TRY Gym programme in equipping participants’ developmental psychosocial competence.

#### 4.2.1. Social Competence

Evidenced by the significant improvement in the social competence scale, the training in this programme is intricately connected to enhancing individuals’ ability to engage in meaningful interactions with others, as it directly addresses the behavioural manifestations of one’s emotional and regulatory competencies in social contexts. By offering knowledge and opportunities to practice empathy, the training helped individuals develop a deeper understanding of others’ emotions and perspectives, which is essential for fostering strong, empathic, and supportive relationships. Additionally, learning to observe and listen to the needs of people further strengthens their social competence, enabling participants to respond appropriately and effectively in social situations. These ways to promote social competences were also elaborated on and examined in some studies [117,118]. As in TRY Gym, interviewees pointed out that they had an increased willingness to engage in short conversations with unfamiliar people, and provide help to people with emotional issues by using listening and communication skills throughout the programmes. These skills are not only critical for navigating complex social environments and promoting better communication among trainees, but also for building positive social roles among adolescents to create a secure, supportive environment with a strong sense of belonging.

#### 4.2.2. Emotional Competence

This significant improvement in emotional competence aligns with findings from similar international interventions aimed at enhancing social and emotional skills among adolescents. For instance, the Mindfulness-Based Education Programme evaluated by Schonert-Reichl and Lawlor [119], which incorporated components such as mindfulness practices, eliminating negative thinking, teamwork, and goal setting, reported positive significant improvements in pre- and early adolescents’ social-emotional competence. This convergence suggests that structured elements focusing on emotional awareness and regulation—shared between TRY Gym’s resilience session (e.g., identifying stress triggers, exploring coping strategies, and reframing negative thoughts) and the mindfulness programme’s emphasis on negative thought elimination and mindfulness—can effectively foster emotional intelligence and adaptive responses to emotions. However, TRY Gym innovates by integrating self-compassion practices alongside resilience training, potentially offering a more holistic approach to emotional well-being, though our nonsignificant self-compassion results indicate this aspect may require further refinement for greater impact.

In terms of resilience, TRY Gym’s significant gains contrast with evidence from a systematic review of universal school-based well-being interventions in Australia by Gunawardena et al. [120], where resilience was assessed in 7 out of 29 programs using instruments like the Connor–Davidson Resilience Scale, The Child and Youth Resilience Measure, and The Resilience Youth Development Module, yet most reported no significant effects. For example, interventions involving outdoor activities (e.g., football or “Wellbeing Warriors” programmes [121,122,123]), behavioural activation with emotional regulation [124], self-efficacy and coping strategies [125], psychoeducation [126], and comprehensive social-emotional skills-building (focusing on emotional literacy, positive coping, problem-solving, stress management, and peer support [127]) largely failed to yield improvements across various resilience domains. This divergence highlights TRY Gym’s potential innovation in its targeted, multi-component resilience session, which not only addresses stress identification and coping flexibility but also emphasizes emotional restructuring through reframing, elements that may have contributed to the observed median increases and participant-reported benefits like acquiring emotional control techniques (e.g., deep breathing) and behavioural changes (e.g., appropriate emotional expression). Such components echo effective aspects of prior programs [128,129], but TRY Gym’s structured progression from self-exploration to practical reframing appears to have overcome common limitations in other interventions, fostering enduring psychological resilience as evidenced in interview responses.

Furthermore, while self-compassion showed only a nonsignificant trend toward improvement in our study, the programme’s self-compassion training—encouraging kindness and mindfulness toward negative emotions—mirrors elements in mindfulness and self-compassion interventions [130,131], which have demonstrated reductions in rumination, self-criticism, and isolation. Post-test interviews revealed participants’ shifts toward constructive self-reflection and reduced self-criticism, suggesting latent benefits that partially support emotional competence gains. Compared to the Schonert-Reichl and Lawlor programme [119], which achieved broader social-emotional enhancements without an explicit self-compassion focus, TRY Gym’s integration of this component represents an innovative extension, potentially preventing mental health issues through balanced emotional processing, though future iterations could intensify these practices to achieve statistical significance, as seen in more targeted self-compassion programmes. Overall, these comparisons contextualize TRY Gym within the landscape of adolescent well-being interventions, underscoring its convergences with established models (e.g., emotional regulation and coping emphases) while highlighting innovations in combining resilience and self-compassion for modest yet promising emotional competence outcomes.

#### 4.2.3. Cognitive Competence

The participants’ cognitive competency increased significantly due to targeted training in listening and micro-counselling abilities. Listening and micro-counselling skills are critical components of cognitive competency. The listening and empathy lessons assisted teenagers in developing these abilities by training them to notice and comprehend the needs of others. Listening and communication exercises taught them to actively listen and reply with understanding, improving their interpersonal skills. This not only increased their ability to provide meaningful support, but also promoted positive relationships. Examples could be seen in participant interviews, where many suggested a greater inclination to communicate with others and, more crucially, a greater proclivity to discuss more sensitive matters with their friends. As teenagers improved in these skills, they established supportive situations critical for their cognitive growth and the mental well-being of their peers.

Furthermore, the programme aimed to help teenagers build effective coping techniques, which were necessary for them to manage and navigate stress, as discussed in transactional theory [132]. The resilience workshops assisted adolescents in recognizing sources of stress and exploring how they can overcome adversity or obstacles. By allowing participants to construct mental health education initiatives (such as mental health quizzes to improve mental health awareness and busking to promote relaxation), the programme encouraged students to think critically and creatively, which they found highly engaging. As a result, the programme’s emphasis on introspection and practice opportunities assisted participants in adapting their ways of thinking and problem-solving in various dynamic circumstances, preparing them to deal more creatively and easily with the obstacles of everyday life.

#### 4.2.4. Self-Competence

The TRY Gym programme significantly enhanced participants’ sense of mastery, indicating improved feelings of control and strongly supporting H1. Self-efficacy competence and awareness of personal strengths showed directional improvements, suggesting potential growth in confidence and self-awareness that partially supports H1.

A key component of the programme was the 24 Character Strengths test, which enabled participants to identify and apply personal strengths such as creativity, leadership, and kindness. This strengths-based approach fostered self-awareness, which prior research links to improved decision-making [133], team performance [134], and leadership skills [135]. By applying these strengths in designing and organizing youth-led activities during the implementation phase, participants reported increased confidence and a stronger sense of identity. These findings align with Shoshani and Steinmetz [136], whose positive psychology intervention similarly emphasized strengths identification and application, yielding significant increases in self-efficacy among adolescents and contributing to enhanced mental health and well-being. TRY Gym’s innovation lies in integrating this strengths-based framework with youth-led initiatives, allowing participants to actively apply their strengths in real-world contexts, likely driving the observed improvements in sense of mastery.

In contrast, Gunawardena et al. [120] reviewed self-efficacy outcomes in Australian school-based well-being interventions, reporting mixed results. Two interventions—one using martial arts [121,122] and another targeting resilience in regional youth [125]—reported small effects on self-efficacy, while an outdoor youth programme showed a medium-to-large effect [137]. TRY Gym’s nonsignificant self-efficacy trend contrasts with these significant findings, possibly due to its broader focus on multiple competencies, which may have diluted its impact on self-efficacy compared to more targeted interventions. However, the significant improvement in sense of mastery aligns closely with the outdoor programme’s stronger effect, likely driven by shared elements like experiential learning through youth-led activities, where participants practiced skills such as brainstorming and project planning.

Qualitative feedback from interviews further supports these outcomes, with participants reporting enhanced confidence in communication and leadership during youth-led activities. These activities fostered a sense of accomplishment, consistent with research linking such engagement to improved mental well-being [138,139,140]. Unlike the interventions reviewed by Gunawardena et al., which often relied on specific modalities (e.g., martial arts or outdoor activities), TRY Gym’s combination of strengths identification with hands-on leadership opportunities represents a novel approach. This integration not only reinforced participants’ belief in their abilities but also equipped them with practical skills for future challenges, positioning TRY Gym as a promising model for fostering self-competence, though further focus on self-efficacy-specific components could enhance outcomes.

#### 4.2.5. Motivational Competence

Although motivational competence was found insignificant in the analysis, participants demonstrated stronger engagement and, more importantly, higher persistence and motivation throughout the programme. By allowing them to design their own mental health-related activities, adolescents were encouraged to think creatively and take ownership of their projects. This autonomy fostered a sense of responsibility and initiative, as they mapped and located well-being alliances within their communities. In the interviews, most participants reported enjoying the programme and some of them recalled unforgettable memories of spending hours with teammates designing and planning games for the presentation at the mega events. The process of brainstorming, planning, and executing their ideas enhanced their problem-solving skills and boosted their confidence. As they navigated the challenges of designing and conducting their activities, adolescents developed a proactive mindset, which was crucial for sustained motivation and personal growth, suggested by other studies in the youth context [141,142]. Furthermore, given that the activities were school-based, preparation often took a long time, as trainees could only work on them after school. Despite these constraints, they completed their tasks, demonstrating remarkable dedication and commitment. Trainees often had to juggle their academic responsibilities with their involvement in TRY Gym, which meant spending lengthy hours in discussions and preparations. These demonstrated a high level of motivation and persistence by participants throughout the programme.

### 4.3. Hypothesis Set 2: Misconceptions, Negative Attitudes, and Stigma Toward Mental Disorders

For H2, the nonsignificant result in the Perceived Devaluation-Discrimination Scale did not support the expected effect of the TRY Gym project on reducing the level of mental health stigma among participants. Observing the U-shape pattern of data, the downward trend between the baseline and post-training stage suggested a short-term effect on reducing misconceptions, negative attitudes, and stigma towards mental disorders among adolescents and youths.

Possible reasons could be drawn from the qualitative findings. During the interview, participants reported gaining more understanding and higher acceptance and less perceived discrimination when facing people with mental health issues during the interview. Participants also reported being interested in the activity of the human library with young people with mental illness to share their experiences and how to cope with and live with mental illness. The human library enhanced more understanding and awareness of mental health issues and reduced their stigmatization, with participants reporting more willingness to help those in need instead of being reluctant to offer help. These encouraging qualitative results suggested a decrease in Perceived Devaluation-Discrimination, further explaining the increase in motivation to create a stigma-free community, which aligned with the results of other studies with human library interventions [143,144]. However, participants’ motivation did not persist long throughout the programme. The primary goal of most youth-led initiatives was to enhance the mental health of teenagers through conducting relaxation or mindfulness activities rather than lessen stigma and discrimination in society. This indicates a limited short-term effect on reducing misconceptions, negative attitudes, and stigma towards mental disorders among adolescents and youths, explaining the inconsistent pattern of reducing the mental health-seeking attitude and Perceived Devaluation-Discrimination Scale.

On the other hand, the inconsistent changes in mental help-seeking attitudes suggested a limited effect on encouraging participants to seek external support for their mental health and psychological issues. Although the human library shared and provided channels and information about mental health-related organizations, participants seemed to be hesitant and reluctant to seek help from professionals. Among all the human libraries shared by different guest speakers, the experience of seeking help from mental health professionals/institutes, especially the first time going into a mental institute, was either not included or briefly mentioned. Understandably, they might not want to talk about the unpleasant experience with participants. However, most of the trainees lack a relevant understanding of this experience and negative attitudes might be imposed, with mental help-seeking behaviours being regarded as serious illnesses. Furthermore, the interview results showed that participants frequently thought that mental health programmes or interventions from organizations had little impact as they were inaccessible and did not cater to the specific needs of each individual. These negative attitudes limited participants’ motivation to find mental health support among participants. Revision should be conducted to examine the content.

A previous systematic study [145] found that contact with experienced individuals with mental disorders, whether in person or through film, increases knowledge and awareness of mental disorders in six studies [146,147,148,149,150,151]. Regarding the same systematic review [145], among the four studies [152,153,154,155] addressing emotional responses toward individuals with mental disorders, only one study [152] included live interaction with individuals with mental disorders in the intervention, which significantly reduced stigma in the intervention group from pre- to post-intervention. The systematic reviews focused on school-based interventions, similar to TRY Gym, that promote mental health literacy and reduce mental health stigma. In contrast, our listening sessions adopt a broader approach, aiming to nurture empathy in participants through enhanced listening and communication skills, applicable across diverse contexts without targeting a specific group, thereby fostering a more inclusive understanding and connection. Therefore, the human library section should enhance knowledge and awareness of mental disorders and reduce stigma. However, our results contradict previous studies, indicating that the relationship between interactions with individuals in recovery from mental disorders and increased knowledge, awareness, and stigma reduction requires further exploration.

### 4.4. Hypothesis Set 3: Overall Mental Well-Being

The TRY Gym programme significantly enhanced adolescents’ mental well-being, supporting H3 by fostering comprehensive development across five key competencies: social, self, emotional, cognitive, and motivational. Socially, the programme cultivated improved communication skills and healthy relationship-building, essential for creating supportive peer networks. In terms of self-competence, it promoted self-awareness and self-efficacy, empowering adolescents to value their unique strengths. Emotionally, it equipped participants with effective coping and self-compassion strategies, helping to reduce anxiety and stress. Cognitively, the programme sharpened critical thinking and problem-solving skills, aiding adolescents in navigating real-life challenges and developing counselling abilities. Motivationally, goal-setting workshops and positive reinforcement fostered sustained personal motivation and persistence. These combined improvements align with findings from Shoshani and Steinmetz [136], whose positive psychology intervention similarly targeted multiple competencies, resulting in significant reductions in psychological distress, depression, and anxiety symptoms among adolescents. TRY Gym’s holistic approach, integrating youth-led activities and strength-based exercises, mirrors this multi-faceted strategy but innovates by emphasizing practical application through participant-driven initiatives, which likely amplified well-being outcomes.

Comparatively, Gunawardena et al. [120] reviewed 29 Australian school-based well-being interventions, with eight measuring well-being using validated instruments. Only one music-based intervention showed a significant medium effect on well-being [156], while others, including whole-school approaches [157], music interventions [158], and positive psychology programs [159,160], reported no significant impact. An Acceptance and Commitment Therapy (ACT) intervention showed a small effect on subjective well-being [161]. TRY Gym’s significant well-being improvements contrast with these mixed results, suggesting its comprehensive design—integrating social, emotional, and motivational components—may be more effective than single-modality interventions like music or ACT. However, like the effective music intervention, TRY Gym’s emphasis on engagement through youth-led activities may have fostered a sense of purpose and community, contributing to its success.

Similarly, a systematic review [162] of mindfulness-based interventions in UK secondary schools, found modest but significant effects on well-being in a feasibility study [163], with sustained outcomes linked to mindfulness practice. While TRY Gym does not explicitly use mindfulness, its emotional competence strategies, such as self-compassion and coping skills, share similarities with mindfulness-based approaches, promoting emotional regulation and stress reduction. TRY Gym’s broader focus on five competencies extends beyond mindfulness, offering a more comprehensive framework that addresses diverse aspects of well-being, potentially enhancing its impact compared to the narrower focus of mindfulness interventions.

The TRY Gym programme’s unique integration of youth-led activities, strengths-based self-awareness, and multi-competency development distinguishes it from interventions reviewed by Gunawardena et al. and Mackenzie and Williams, which often focused on singular approaches. This holistic model likely contributed to its robust well-being outcomes, as participants reported practical applications of skills in real-world settings, fostering a sense of accomplishment and community. However, the nonsignificant results in some areas, such as self-compassion, suggest that further refinement of specific components could strengthen outcomes, aligning with findings from mindfulness studies that emphasize sustained practice for greater impact [163]. Collectively, these improvements created a holistic support system, resulting in a marked enhancement in overall mental well-being, positioning TRY Gym as a promising model for adolescent mental health interventions.

### 4.5. Limitations

First, the study’s non-randomized, pre-post design without a control group limits causal inferences, as external factors (e.g., school environment or maturation effects) could influence outcomes. This is a common challenge in community-based interventions, as noted in similar peer-led programs like Sources of Strength [164].

Second, the sample size (*n* = 80) and focus on Hong Kong secondary schools restrict generalizability, particularly to diverse cultural or socioeconomic contexts. Self-reported measures, while practical, are susceptible to response biases, potentially inflating perceived improvements in areas like emotional competence.

Third, since the quantitative research highly relied on the self-report survey, which contains over 100 items for each test, the lengthy test could cause response fatigue, which might affect the quality of the data. Considering the aforementioned limitations, the results should be interpreted with caution.

Additionally, the lack of long-term follow-up assessments means we cannot confirm the sustainability of gains, especially for nonsignificant outcomes like stigma reduction, which showed a U-shaped pattern indicative of short-term effects waning. Future iterations should incorporate randomized controlled trials, objective measures (e.g., behavioural observations), and diverse samples to strengthen evidence. Despite these limitations, the mixed-methods approach (combining scales, interviews, and feedback) provides a robust triangulation of data, highlighting TRY Gym’s practical impact.

Although we strive to minimize the involvement of adult facilitators during the implementation phase, the school’s approval process may undeniably restrict students’ freedom. However, this review is essential, as “do no harm to participants” is a fundamental principle in research and a core responsibility of the school. Furthermore, we believe that the professionalism of social workers can support student-led initiatives, empower students, and minimize the impact of power asymmetries on their planning and execution to the greatest extent possible. Nevertheless, when students lack ideas or motivation, social workers inevitably offer suggestions to inspire them—a phenomenon observed in some schools. The inability to fully address the constraints imposed by institutional hierarchies on authentic youth leadership, and to navigate power asymmetries between adult facilitators and youth participants may have limited the effectiveness of this study. Nevertheless, the research findings retain significant reference value. Future research could explore ways to mitigate the constraints imposed by institutional hierarchies on authentic youth leadership and address power asymmetries between adult facilitators and youth participants.

### 4.6. Future Implications

Despite these limitations, the study still highlights a transformative, resilient-promoting, and youth-driven prevention programme designed for and led by the youth. In the post-COVID era, prevention programmes should not just provide mental health information and knowledge for young people but also develop opportunities for them to proactively take part in the programmes. For the adolescents in the high-pressure education system, the programmes might affect the community and the younger generation’s mental health in addition to promoting personal development. Future work could incorporate youth-generated conceptions of well-being, especially those beyond Western psychological constructs. Although not all results in our findings are significant, applying TRY Gym in schools could be effective in promoting mental well-being and some psychosocial competencies for students. It could serve as a valuable tool for the government and schools to alleviate the concerning mental health issues among adolescents and the high student suicide rate, while also buying time to implement reforms that address the root causes of these issues.

## 5. Conclusions

In summary, the programme was not only successful in enhancing the trainees’ mental health but also empowered them in terms of knowledge and abilities to create a mental health-supportive society. Six of the twelve measuring scales demonstrated statistically significant positive effects in social, emotional, cognitive, and self-competence, as well as resilience and mental well-being. With a variety of themes covered, the training sessions were effective in imparting important knowledge about mental health issues and providing plenty of practice opportunities for participants to learn and apply the acquired techniques in everyday life situations. Regarding the unexpected results, efforts will be made to review the design and execution of the programmes to increase the TRY Gym project’s implementation fidelity and effectiveness. Through adding additional practices in the implementation phase and revising topics discussed in the training sessions, we can anticipate more profound effects in other outcome measures (sense of mastery, awareness of personal strength, self-compassion, and self-compassion), along with less perceived discrimination and a more positive attitude toward mental health help-seeking behaviours. Overall, the favourable outcomes of the first-year TRY Gym programme demonstrated the effective key features—transformative, resilient, and youth-led.

## Figures and Tables

**Table 1:** Details of eight sessions of the TRY Gym in the training stage.

Session	Topic	Goals	Contents
1	Introduction to Mental Health	Know about Situation and Knowledge of Mental Health	Know how to identify symptoms of mental/mental disorders
2	Recovery	Reduce Stigma and Stereotypes about Mental Illness	Human Library (Recoveree sharing live experiences)
		2. Know about Mental Health Recovery	
3	Resilience	Identify the Source of Stress	Practice changing negative mindsets (ABCDE Model)
		2. Explore How to Overcome Obstacles or Difficulties	
4	Self-Compassion	Know about Self-compassion	Practice self-compassion skills (Mindfulness, Common Humanity, and Self-kindness)
		2. Practice Self-compassion and Improve the Ability	
5	Listening	Know about and Practice Empathy	Practice listening and communication skills (Active-Constrictive Responding)
		2. Learn to Observe and Listen to Other People	
6	Personal Strength	Discover Their Personal Strength (24 Character Strengths)	24 Character Strengths Test
		2. Know the Elements to Design a Mental Health Educational Programme	Know how to design a programme
			Start to design a mental health programme
7	Community Resources	Know and Search Available Services and Resources in the Community	Know about LevelMind@JC Know about formal and informal services Know about services provided by various organizations
8	Use Social Media to Promote Mental Health	Know about the Opportunities and Challenges of Using Social Media to Promote Mental Health	Human Library
			Review personal growth
		2. Review and Conclusion 3. Prepare for the Next Phase	Preview of next stage

**Table 2:** Demographics (*n* = 80).

Demographic	Mean	SD	*n*	%
Age	16.05	0.953		
Gender				
Male			37	46.3
Female			43	53.8
Secondary				
Secondary 3			11	13.8
Secondary 4			44	55.0
Secondary 5			25	31.3
Monthly Household Income				
HKD 0–10,000			3	3.8
HKD 10,001–20,000			9	11.3
HKD 20,001–30,000			5	6.3
HKD 30,001–40,000			2	2.5
HKD 40,001–50,000			4	5.0
HKD 50,001–60,000			1	1.3
HKD 60,001–70,000			1	1.3
HKD 70,001–80,000			0	0
HKD 80,001 or above			6	7.5
Not Sure			49	61.3
Residential Housing Type				
Public Housing			24	30.0
Home Ownership Scheme (HOS)			12	15.0
Private			38	47.5
Others			6	7.5
Location of School				
Hong Kong			19	23.8
Kowloon			29	36.3
New Territories			32	40.0
Organization				
CYS			32	40.0
TWGHs			13	16.3
SKHWC			24	30.0
SJS			11	13.8

**Table 3:** Results of Kolmogorov–Smirnov ^a^ Test in psychological outcomes (*n* = 80).

Scales	Baseline			Post-Training			Implementation		
	Statistic	df	Sig.	Statistic	df	Sig.	Statistic	df	Sig.
Social Competence Scale	0.117	80	0.008	0.132	80	0.001	0.116	80	0.010
Emotional Competence	0.086	80	0.200 *	0.103	80	0.035	0.059	80	0.200 *
Cognitive Competence	0.066	80	0.200 *	0.095	80	0.070	0.175	80	<0.001
Self-Efficacy Competence	0.068	80	0.200 *	0.089	80	0.177	0.149	80	<0.001
Resilience	0.064	80	0.200 *	0.096	80	0.068	0.101	80	0.043
Motivational Competence	0.125	80	0.003	0.108	80	0.022	0.160	80	<0.001
Sense of Mastery	0.123	80	0.005	0.153	80	<0.001	0.146	80	<0.001
Perceived Devaluation-Discrimination	0.094	80	0.076	0.205	80	<0.001	0.185	80	<0.001
Mental Well-being	0.067	80	0.200 *	0.109	80	0.020	0.104	80	0.033
Self-Compassion	0.145	80	<0.001	0.139	80	0.001	0.160	80	<0.001
Awareness of Personal Strength	0.117	80	0.008	0.111	80	0.016	0.115	80	0.011
Positive Attitudes towards Seeking Services	0.068	80	0.200 *	0.167	80	<0.001	0.114	80	0.012

* This is a lower bound of the true significance. ^a^ Lilliefors Significance Correction.

**Table 4:** Median (MDN), Friedman and post hoc test results (df = 2) of psychological outcomes (*n* = 80).

Scales	Baseline MDN (T_1_)	Post-Training MDN (T_2_)	Implementation MDN (T_3_)	Friedman Test χ2	Benjamini–Hochberg Adjusted *p*	Post Hoc Pairwise Comparison *		
						Pairwise Comparison	r (Effect Size)	Bonferroni Correction Adjusted *p*
Social Competence	28	30	30	20.216	<0.001	T_1_–T_2_	0.41	0.001
						T_1_–T_3_	0.38	0.002
Cognitive Competence	27	29	29	13.251	0.003	T_1_–T_2_	0.33	0.009
						T_1_–T_3_	0.33	0.009
Emotional Competence	24	26	26	9.017	0.022	T_1_–T_3_	0.30	0.024
Resilience	26.5	29	29	16.050	0.001	T_1_–T_2_	0.41	0.001
						T_1_–T_3_	0.30	0.024
Self-Compassion	48	48	48.5	1.199	0.732			
Self-Efficacy Competence	29	30.5	31	5.062	0.136			
Sense of Mastery	18	19	19.5	16.572	0.002	T_1_–T_2_	0.31	0.015
						T_1_–T_3_	0.40	0.001
Awareness of Personal Strength	50	51	51	0.164	0.921			
Motivational Competence	37	38	37	0.433	0.967			
Mental Well-being	24	23	26	14.676	0.002	T_1_–T_2_	0.39	0.002
						T_2_–T_3_	0.30	0.022
Positive Attitudes towards Seeking Services	9.5	8	9	0.351	0.915			
Perceived Devaluation-Discrimination	38	36	37	4.252	0.179			

* Only significant results would be reported for pairwise comparison post hoc test.

**Table 5:** Descriptive statistics for participants’ opinions.

Item	Post-Training Phase		Post-Implementation Phase	
	Mean	SD	Mean	SD
The programme has helped me understand the importance of recognizing and being aware of mental health.	4.16	0.834	4.21	0.833
The programme has helped me understand the importance of promoting community awareness of mental health.	4.12	0.880	4.24	0.757
The programme has helped me understand the emotional needs and feelings of those around me, making me more empathetic towards them.	4.10	0.872	4.28	0.79
The programme has helped me better understand how to seek suitable resources and methods to support individuals in need of mental health support, including myself.	4.05	0.867	4.24	0.757
Overall, I feel that the programme has been helpful to me.	4.19	0.907	4.28	0.854

## Data Availability

The raw data supporting the conclusions of this article will be made available by the authors on request.
